# Prevention, diagnosis, and management of donor derived infections in pediatric kidney transplant recipients

**DOI:** 10.3389/fped.2023.1167069

**Published:** 2023-04-21

**Authors:** Katrina Epperson, Clarkson Crane, Elizabeth Ingulli

**Affiliations:** Department of Pediatrics, Division of Pediatric Nephrology, University of California at San Diego and Rady Children’s Hospital, San Diego, CA, United States

**Keywords:** donor derived infections, pediatric kidney transplant, donor evaluation, unexpected donor derived disease, prevention

## Abstract

Donor derived infections (DDIs) in pediatric kidney transplant recipients remain challenging to diagnose and can result in serious morbidity and mortality. This review summarizes the current guidelines and recommendations for prevention, diagnosis, and treatment of unexpected DDIs in pediatric kidney transplant recipients. We provide a contemporary overview of DDI terminology, surveillance, epidemiology, and recommended approaches for assessing these rare events with an emphasis on the pediatric recipient. To address prevention and risk mitigation, important aspects of donor and pediatric candidate evaluations are reviewed, including current Organ Procurement and Transplantation Network (OPTN) and American Society of Transplantation (AST) recommendations. Common unexpected DDI encountered by pediatric transplant teams including multi-drug resistant organisms, tuberculosis, syphilis, West Nile Virus, toxoplasmosis, Chagas disease, strongyloidiasis, candidiasis, histoplasmosis, coccidioidomycosis, and emerging infections such as COVID-19 are discussed in detail. Finally, we consider the general challenges with management of DDIs and share our experience with a novel application of next generation sequencing (NGS) of microbial cell-free DNA that will likely define a future direction in this field.

## Introduction

Kidney transplantation remains the treatment of choice for children with advanced or end-stage kidney failure. However, transplantation is not without risk. Balancing immunosuppression with risk of infection remains an ongoing challenge, especially in the context of donor derived infections (DDIs). DDIs can cause serious morbidity and mortality for solid organ transplant (SOT) recipients. These complications can be even more devastating in pediatric SOT recipients and early recognition, timely reporting, close monitoring, and appropriate management are essential to help mitigate these risks.

Unexpected donor derived disease (DDD) was recently reported to occur in 0.18% of SOT recipients in a review of patients from 2008 to 2017 (1). Unexpected DDD is most commonly due to an infectious pathogen, but also malignancies, allergic disease, and/or metabolic disease are reported (1). In this review, we will focus on DDIs, provide a background for defining DDIs, survey the current epidemiology of DDIs, review ways to mitigate DDI risk while maximizing organ utilization, and summarize recommendations for evaluating and managing pediatric kidney transplant candidates and potential donors for DDI risk.

## Definitions

Since 2005, all suspected DDD transmission events are required to be reported to the Organ Procurement and Transplantation Network (OPTN), as outlined in policy 15.4 (2). DDD includes infections, malignancy, and other potentially transmissible conditions (3). With regard to infections, DDIs are often characterized as “expected” or “unexpected.” Expected infections include those in which a donor is previously known to be infected with a pathogen that can be detected and treated with commonly available therapies or mitigation strategies (4). Examples include Cytomegalovirus (CMV), Epstein-Barr virus (EBV), BK polyomavirus (5). In many adult centers, donor-transmission of Hepatitis C Virus (HCV) could be considered in the “expected” category in the setting of intentional transplantation from HCV positive donors into HCV negative recipients given the availability and excellent efficacy of post-transplant antiviral treatment (6–8). Of note, this not a common practice in pediatrics, especially in pediatric kidney transplants.

In contrast, “unexpected” transmission of various types of pathogens from donor to recipient occur when donor infection is previously unknown, undetected, and/or incompletely treated (5). For the purpose of this review, we will focus on unexpected DDIs. There are many potential pathogens that have been reported to be transmitted *via* SOT and it remains essential for clinical transplantation teams to remain vigilant to this risk, react quickly to potential DDI, and start treatment immediately.

## Surveillance and current epidemiology

**Figure 1:**
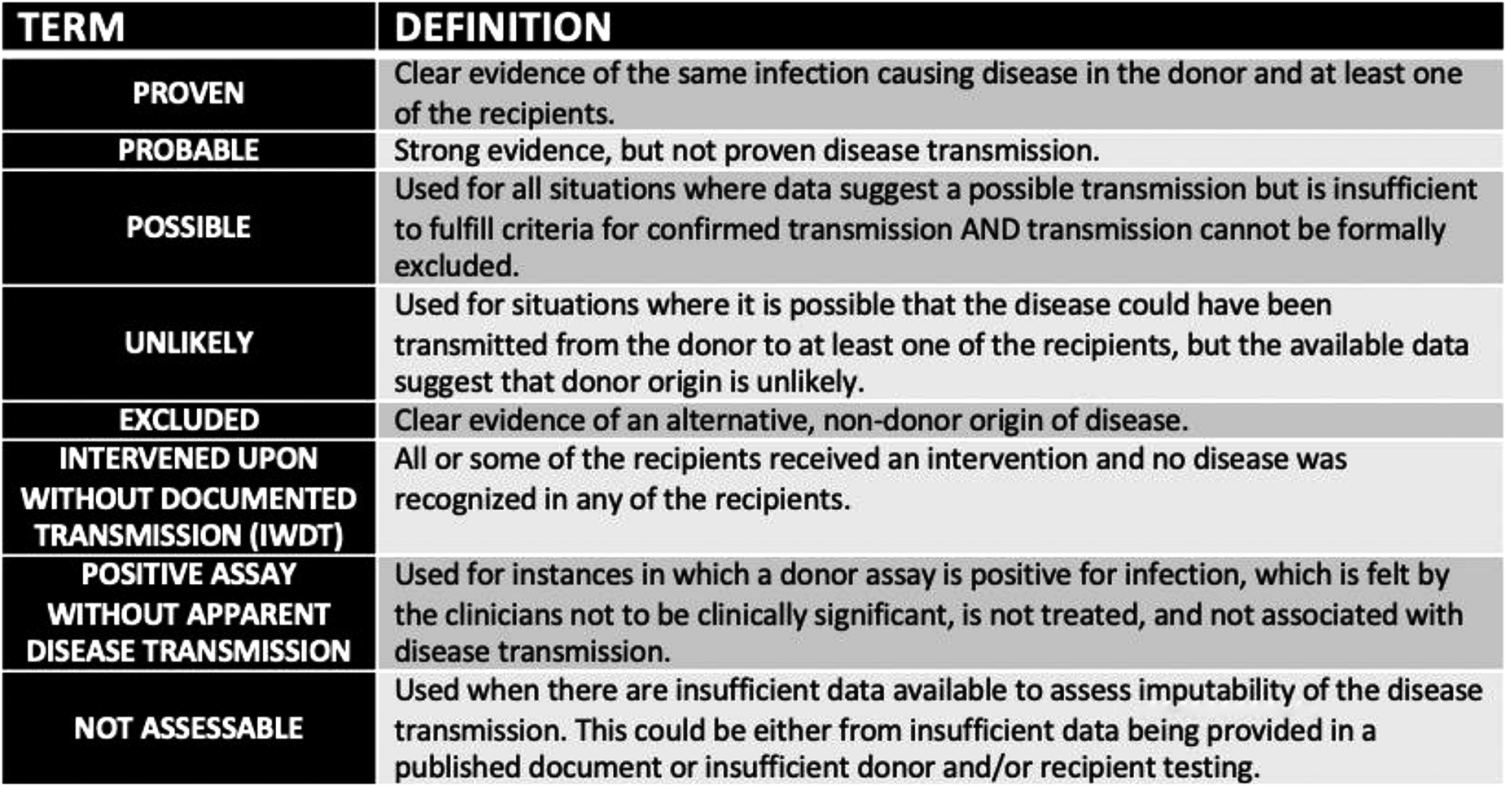
Terms used to describe potential donor derived transmission events.

The Ad Hoc Disease Transmission Advisory Committee (DTAC) reviews any potential donor derived transmission events in a blinded fashion. After committee review, the probability of a transmission event is determined and classified as proven, probable, possible, unlikely, excluded, intervened upon without documented transmission (IWDT), positive assay without apparent disease transmission, or not assessable as defined in Figure 1 (9). While reporting of potential events is required by OPTN policy 15.4, it should be noted that it is done on a voluntary basis and there is no active surveillance program, which could result in under-reporting of suspected transmission of DDD (1).

The most recent published report from DTAC describes all reports received between 2008 and 2017 in both pediatric and adult SOT donors and recipients. Of the 2,185 reports during that time period, 335 (15%) were considered a proven or probable donor transmission event with an overall rate of unexpected donor derived disease at 0.18% of all transplant recipients (1). The majority (67%) were infections and rates of graft loss and mortality in recipients with DDI were 12% and 15%, respectively (1). Of note, in another prospective analysis of the Spanish Research Network for the Study of Infection in Transplantation, it was found that DDI was slightly higher at 1.7% with 40% mortality from the acquired infection (10).

A prior report of DDD limited to pediatric donors and recipients showed overall similar results. Between 2008 and 2013, there were 103 reported potential DDIs reported to involve pediatric donors or recipients. With regard to pediatric recipients, 11 cases were classified as proven or probable events (11). Infection was responsible for 9 of the 11 cases with three viral, two bacterial, three parasitic, and one fungal infection being identified (11). There were two deaths in this group, giving an attributable death rate of 0.02% of all pediatric SOT recipients (11).

## Candidate evaluation

All pediatric kidney transplant candidates need to be screened for infectious diseases as part of their pre-transplant work-up. Candidate screening directs the pre-management of known infectious disease complications and risk management of DDIs. Furthermore, always taking into consideration the underlying etiology of pediatric kidney failure along with previous and/or current immunosuppression will help clinicians consider a candidate's risk of DDIs, both expected and unexpected. At our center, all our patients and their families have a visit with an infectious disease specialists as part of the initial evaluation and they receive counseling prior to transplant. We recommend infectious disease consultation for all patients prior to transplantation.

The candidate's pre-transplantation infectious disease evaluation has been developed by multiple organizations and oversight groups. All testing must be performed in a Clinical Laboratory Improvement Amendments (CLIA)-certified laboratory or equivalent. For a candidate to be eligible for an organ transplant, the following testing is required (unless testing would violate state or federal laws) by OPTN per policy 15.2, updated in 2020 (12):

- Human Immunodeficiency Virus (HIV) using United States Centers for Disease Control and Prevention (CDC) recommended HIV laboratory algorithm
- Hepatitis B Virus (HBV) surface antigen (HBsAg), hepatitis B core antibody (total anti-HBc), hepatitis B surface antibody (HBsAb)
- Hepatitis C Virus (HCV) antibody (anti-HCV), hepatitis C ribonucleic acid by nucleic acid test (NAT).

As of July 1, 2022, the OPTN updated its policy on pre-transplant evaluation for all candidates less than 12 years of age with regard to HIV, HBV, and HCV testing. Pediatric patients less than 12 years old must be tested any time prior to transplant and the testing must be documented, but it does not have to occur at time of hospital admission for transplant. This policy change was made given incidence of these infectious are very low in this age group and there is a large concern for overdrawing blood, especially immediately prior to a solid organ transplant (13).

In addition to the required infections disease tests by OPTN, the most recent American Society of Transplantation (AST) guidelines from 2019 and KDIGO Clinical Practice Management Guideline on Evaluation and Management of Candidates for Kidney Transplantation 2020, recommend the following candidate infectious disease screening:

- Screening for periodontal disease and treatment prior to transplant
- Tuberculosis (TB) with purified protein derivative (PPD) or interferon-gamma release assay and chest x-ray
- CMV IgG antibody
- EBV with EBV viral capsid antigen IgG, IgM and/or EBV nuclear antigen (EBNA) IgG
- Herpes simplex virus IgG
- Varicella IgG
- Mumps IgG, Rubella IgG, Rubeola IgG
- Human T-lymphotropic virus 1 & 2 with IgG serology in candidates from endemic areas
- Syphilis with any of the following: treponema pallidum particle agglutination, treponema pallidum immunoassay, rapid plasma regain, venereal disease research laboratory
- Toxoplasmosis with IgG antibody

In endemic areas, other testing should be strongly considered such as strongyloides serologies, coccidiodes serologies, histoplasmosis serologies/urine antigen tests, chagas serologies, malaria screening with a malaria blood smear, West Nile Virus serologies or NAT, and/or hepatitis A total antibody, hepatitis A IgM (4, 12). This list is not all inclusive and we would recommend infectious disease consultation for further recommendations on endemic infectious risks and testing pre-transplant.

The Coronavirus Disease 2019 (COVID-19) pandemic presented new challenges for infectious disease screening and recommendations are evolving. Based on a review from 2021, the current recommendation is to wait at least 4–6 weeks after recovering from COVID-19 prior to transplantation. It is also recommended that patients undergo pulmonary function testing (PFT) and have SARS-CoV-2 IgG and RT-PCR once fully recovered (14). If a candidate continues to have positive RT-PCR after full clinical recovery, we recommend consultation with infectious disease prior to proceeding with transplantation.

All eligible vaccinations should be administered to patients on the waiting list potentially on an accelerated schedule, if necessary. A 4-week delay of transplantation is recommended after any live vaccinations (4, 12). Many pediatric centers require candidates be fully immunized prior to transplantation, including for SARS-CoV-2, which we also believe is prudent to minimize post-transplantation risks for pediatric kidney transplant recipients.

## Donor evaluation

While it is impossible to eliminate the potential for transmission of DDIs, a thoughtful and thorough approach to donor evaluation can mitigate risk. Various screening protocols have been proposed, although the sensitivity and specificity of these approaches are unknown (15). In general, it is ideal to (1) carefully review the donor's medical and social history, (2) clinically assess the donor and donor organs, and (3) review screening tests for donor infectious diseases. We also recommend all living donors are referred for infectious disease evaluation and counseling prior to donation.

### Donor medical and social history

Specific details from the donor medical and social history can provide insight into the risk of DDI transmission. In the case of a deceased donor, the cause of death should be a starting point. For example, a donor who dies from trauma is a much lower infectious risk than one who died from complications of intravenous drug use (16). Alternatively, a donor who was a drowning victim may become infected with unusual molds, although the exact risk of infection and potential for transmission is unknown (17). The length and details of the donor's terminal hospitalization can also be revealing. Longer hospitalizations, need for dialysis, prior hemopoietic stem cell transplant, and exposure to antibiotics with a narrow gram-negative spectrum are associated with increased rates of multi-drug resistant organisms (MDRO) in donors (16).

Details in a social history can help provide risk stratification, although the reliability of this information can be variable in between living and deceased donors. In the setting of a deceased donor, the social history may be limited and/or incomplete. Elements of a social history that suggest increased risk of blood borne viral pathogens include high risk sexual contacts (persons who have had sex with a person known or suspected to have HIV, HCV, or HBV); men who have sex with men; persons who have had sex in exchange for money or drugs; and persons who have had sex with a person who uses injection drugs nor non-medical reasons), birth to a mother infection with HIV, HBV, or HCV (for donors under 2 years old), persons use injection drugs for non-medical reasons, inmates of a correctional facility for more than 3 days in the past 12 months, persons treated for gonorrhea, chlamydia, or genital ulcers, or persons who have been on hemodialysis in the preceding 12 months (18). Donors with these characteristics are considered Public Health Service (PHS) increased risk donors and additional consent is needed from a recipient prior to transplantation (19).

While use of organs at increased risk of infection transmission at adult centers has the potential to decrease wait times, use of PHS increased risk organs in pediatric recipients is uncommon (20). The pediatric studies that have looked at the use of increased risk donors pediatric kidney transplantation have shown there is no difference in overall mortality and graft survival and are likely underutilized. Clinicians should carefully consider use of an increased risk donor in pediatric candidates, especially given pediatric patients on dialysis have a six time higher rate of mortality than those with a functioning graft (21, 22). Other aspects of a donor medical history that might suggest an increased infection risk include previous infections, vaccination status, potential occupational exposures, blood product transfusion, tattoos and body piercing, and travel history to areas where certain infections may be endemic (15). We advocate for extremely careful consideration prior to accepting a high risk deceased donor kidney for a pediatric patient.

### Clinical assessment of donor and donor organ

Any available information about the clinical and physical assessment of the donor needs to be assessed. This can be done by review of available records (such as imaging studies) or direct observation of the donor and organ by a procurement team. Physical evidence of infection includes unexplained rash, abscesses, ulcers and lymphadenopathy. Recent injection drug use can be inferred by the presence of track marks or skin-popping ulcerations. Cross-sectional imaging can also reveal evidence of active infection, such as metastatic foci, lymphadenopathy, or granulomatous disease (4).

### Infectious disease screening tests for donors

The review of infectious disease-related laboratory data is of utmost importance. OPTN policy requires that all donors undergo testing of commonly transmissible infectious diseases (23). In addition to blood and urine cultures, required testing includes HIV, HBV, HCV, CMV, EBV, syphilis, and toxoplasma. Tests must be performed with FDA approved tests in CLIA-certified laboratories or equivalent. Donor samples for HIV, HBV, and HCV must be obtained within the 96 hours prior to organ procurement. Many other countries have similar testing requirements in place (24).

In deceased donors, all microbiological culture data should be reviewed and ideally a minimum of 48 hours of no culture growth should be available prior to the acceptance of an organ. Ideally, any known active bacterial or fungal infection in the donor should be treated and resolved before transplantation (4). The ability to treat and confirm resolution of infection and/or clinical improvement obviously varies with living or deceased donors. In each case, clinicians should carefully consider the urgency of transplantation. If it is determined that the risk of post-transplantation infection is manageable and the recipient provides informed consent, all efforts should be made to treat the infection pre-transplant and decrease risk of transmission. If the donor has bacteremia, appropriate treatment of the infection for at least 24–48 hours prior to organ procurement is recommended (4).

In the case of bacterial meningitis, there are reports of successful transplantation in adults. At a very minimum, bacterial meningitis should be treated for at least 24–48 hours along with signs of clinical improvement prior to procurement. If the source of the bacterial meningitis is highly virulent, this is a contraindication to transplantation. Encephalitis, especially those of unknown etiology, is also a contraindication for transplantation given risk of transmission (4). Furthermore, we advocate for extremely careful consideration prior to acceptance of any donors with meningitis in the pediatric kidney transplant patient.

Testing of donors for Tuberculosis (TB) is not routinely done for decreased donors due to the length of time required for traditional screening methods. Use of the purified protein derivative (PPD) test is not practical due to the time needed to assess a response (24). Even if there was sufficient time, interpretation of a PPD in a deceased donor can also be complicated by acute and/or chronic illness, treatments or other interventions that the donor may have received, previous latent TB infection, and/or previous BCG vaccination (25). Furthermore, interferon-gamma release assays also present logistical challenges as the test is often sent to a reference laboratory and can take an impractical amount of time to for the assay to run. In this context, the medical and social history is used to screen for TB risk factors. Donors living or traveling to endemic areas, experiencing homelessness, using illicit drugs or have history of recent incarceration should be screened for active disease (26). If active disease is suspected, organs from the donor should not be utilized (27). Any living donors with potential TB risk factors should undergo screening with either PPD or interferon gamma release assay (26). Unexpected DDI from TB will be discussed later in this review.

As noted in the candidate evaluation, COVID-19 presents new challenges for infectious disease screening and recommendations are evolving as new variants arise, changes in therapeutic options, and vaccination availability. SARS-CoV-2 NAT positive organs had similar outcomes compared to COVID-19 NAT negative donors on 30-day patient and graft survival (28). More information regarding SARS-CoV-2 positive donor selection is needed and this this will help inform future guidelines and recommendations. Considering COVID-19 disease severity, imaging findings, history of complications such as ARDS, AKI, and thrombosis would also inform safety of organ for transplantation (28). We would still recommend very careful evaluation of the donor and urgency of transplantation, especially prior to acceptance in a pediatric patient.

Additional considerations for pre-transplant deceased donor testing includes testing for endemic infections such as strongyloidiasis, coccidioidomycosis, histoplasmosis, chagas disease, malaria, West Nile, and/or hepatitis A (12, 29).

### Limitations to infectious disease screening in donors

It is important to consider the limitations of infectious disease screening in donors. There are limitations to diagnostic tests and donor history, especially in the time-sensitive setting of a deceased donor offer. There is always risk of undetected infection for transplant candidates and recipients. Understanding some of these limitations can help inform risk and management of DDIs.

Currently, either serologic or nucleic acid testing (NAT) can be performed to detect viral infections. Historically, much of this evaluation was done using serologic testing given it is less expensive. However, NAT is now being used more commonly due as the technology is becoming more affordable and available.

**Figure 2:**
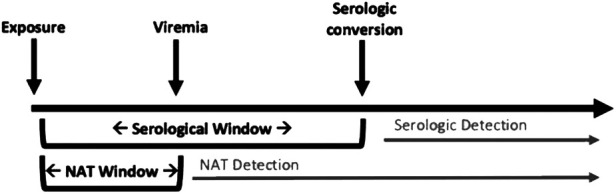
Viral infection and detection by nucleic acid testing (NAT) and serology.

Serologic testing is limited by poor sensitivity early after initial infection, as seroconversion may not occur during the acute phase of an infection. This “window period” can result in a false-negative result and be a high risk period for donor transmission (15). NAT mitigates some of this risk, as these assays are able to detect a pathogen's DNA before an individual will seroconvert. However, there is also a period of time at which the pathogen is at below detectable levels in the blood, termed the “NAT window.” This is depicted in Figure 2 (4). The NAT window is considerably shorter than the serologic window period (5–6 days for HIV NAT compared to 17–22 days for HIV serology) (30), but still merits awareness of the potential for false negative when considering donor offers. As an example, transmission of HCV from nonviremic donors to recipients has been reported in adult patients (31, 32). Where there are no reports of similar occurrences in pediatric recipients, this remains a potential risk and highlights the importance of a thorough donor evaluation and careful attention in the post-operative period.

The potential for hemodilution in deceased donors is another important limitation of NAT or serologic tests (33). Many potential organ donors are victims of trauma and have received significant amounts of blood products or colloid for resuscitation. This can result in false negative tests and care should be taken in interpreting results when donors have undergone massive transfusion. Some of these patients may be considered high risk donors. In addition to mitigating risk by obtaining serial tests, the degree of hemodilution can be estimated and transfusion medicine specialists can also provide guidance (33).

## Unexpected donor-derived infections

There can be a wide spectrum of presentation of unexpected DDIs and clinicians need to have a high index of suspicion. It is possible that DDIs are an under recognized cause of morbidity and mortality (4). Early recognition, timely reporting, close monitoring, and appropriate management are essential to help mitigate the risk of DDIs with pediatric kidney transplantation. While there are many potential unexpected DDIs, we will focus on a few of the more common infectious risks encountered by transplant physicians today.

The American Society of Transplantation Infectious Disease Community of Practice has published general guidelines for the approach to donor-derived infection evaluation in 2019. These guidelines outlined in below involve steps for recognizing potential infections, reporting and communicating, how to collect and retain specimens and data, and tests and management (4).

Recognize Potential Infections
  Consider DDIs in the following settings:
  - Recipients with atypical post-transplant course
  - Recipient with early post-transplant bacterial or fungal infection
  - Multiple recipients of organs from the same donor with the same infection
  - Atypical infection for the early post-transplant period
Report and Communicate
  As soon as any team member considers a potential DDI, a report should be made to all involved organ procurement organizations (OPOs), transplant centers, and transplant authorities:
  - Team should NOT wait until donor origin is confirmed.
  - Report should be made NO later than 24 hours after initial concern.
  - In the USA, reports should be made to UNOS via the Patient Safety Portal and to the patient safety contact for the transplant centers and OPOs. The patient safety contact is listed in DonorNet.
  - Inform all key members of the team, including but not limited to the transplant team, risk management, and media relations.
  - Make direct contact with any involved patients to ensure they are asymptomatic and current clinical status must be obtained.
  - Provide timely updates and information to your patients.
Collect and Retain Specimens and Data
  As soon as you are informed of the potential disease transmission event, collect the following key specimens and data:
  - Contact pathology – retain any fresh residual specimens, biopsies, and/or discarded tissue from the donor and recipient.
  - Contact microbiology – retain any cultures and/or residual specimens that were sent for testing.
  - Contact molecular diagnostics lab – retain any sequences and/or specimens sent for PCR or similar testing.
  - Contact HLA – retain any donor and/or recipient blood, lymph nodes, cells or tissues, particularly pre-transplant specimens.
Test and Manage
  Perform appropriate testing of the recipient for potential DDI:
  - For most viral pathogens you will need direct testing of the virus with PCR/NAT or antigen testing.
  - Serologies may be unreliable in post-transplant period, particularly if there was significant blood transfused.
  - Provide appropriate therapy based on presumed pathogen.
  Continue routine monitoring for disease transmission.

### Unexpected positive cultures, including multi-drug resistant organisms (MDROs) from time of procurement

While every effort is made to treat any known positive cultures prior to transplantation, given the urgency of donation at times, cultures can become positive after transplantation. If a documented bacteremia is found, the recipients should also receive a course of antibiotics targeting the pathogenic organism for at least 7–14 days (4).

A review in a small cohort of urine-only positive donor cultures in adult kidney transplant recipients and preemptive antimicrobial therapy found that preemptive antibiotics did not seem to impact transmission events or outcomes. One hypothesis for this finding was that low transmission rates may be due to Pneumocystis pneumonia (PCP) prophylaxis which could theoretically also prevented transmission. More studies are needed to understand the risk of positive urine cultures for kidney transplant recipients. Regardless, at this time it is still recommended to treat positive urine culture, especially of virulent or more resistant organisms (34).

Special attention must be paid to any multi-drug resistant organism (MDRO) identified in the potential donor as the risk mitigation is extremely difficult and/or unmanageable (4). MDROs are increasing across the world and remain a significant risk, especially to the immunosuppressed SOT population. Previous reports have found that ∼15% of deceased donors have a MDRO on peri-procurement cultures (35).

In a multicenter, retrospective, cohort study of 658 adult SOT recipients with deceased donors, it was found that MDRO significantly increased early post transplantation infection risk, although did not appear to affect long term graft or recipient survival the 1-year mark (35). In this cohort, there were 31 patients with probable DDI and 9 were due to MDROs such as methicillin-resistant staphylococcus aureus, vancomycin resistant enterococci, extended-spectrum cephalosporin-resistant organisms, and Candida glabrata. The MDROs causing DDIs were originally cultured from the donor's respiratory tract (6, 19% of DDIs), blood (1, 3% of DDIs), and perfusate fluid (2, 6% of DDIs). Early identification of MDRO with prompt initiation of antimicrobial therapy in conjunctive with infectious disease consultation can help mitigate the risk of DDIs (37).

### Tuberculosis

Tuberculosis (TB) is caused by the bacterial pathogen, *Mycobacterium tuberculosis.* The risk of DDI with TB varies depending on geographical location. In non-endemic areas, risk factors for donor derived TB include donors who were born outside the US or prolonged residence outside of the US (especially in an endemic area), history of incarceration, homelessness, alcohol and/or substance abuse, healthcare worker, and known history of TB/latent TB infection (LTBI) (36). Active tuberculosis is considered a contraindication to transplantation. LTBI can be considered when weighing the risks and benefits of transplantation or staying on the waiting list (4).

As noted in the donor evaluation portion of this review, it remains challenging to screen for TB given the time limitations of deceased organ donation typically does not allow for PPD placement and there are challenges with interferon gamma release assay. Interferon gamma release assay most often does not result until post-transplantation and had indeterminate results at least 30% of the time (25).

The median time to presentation of donor derived TB in kidney transplant recipients was 2 months post-transplantation and carries a high risk of morbidity and mortality. In a review from 2018 on adult SOT, graft loss was seen in 18.2% of patients, all-cause mortality was 25%, and specifically TB attributable mortality was 44%. The clinical presentation is usually persistent fever and often has an insidious course, which requires a high index of suspicion for diagnosis. Allograft involvement is common in non-lung SOT, presentation commonly included extrapulmonary TB or disseminated TB presentations (25).

Prophylactic treatment with isoniazid is recommended to all organ recipients who received an organ from a donor with LTBI as soon as this risk is identified in the setting of DDI. Current recommendations for duration of prophylactic therapy range from 9 months to 1 year (37). Despite prophylaxis, SOT are still at risk of developing TB and should be monitored carefully (38). If it is determined that a patient has donor derived TB, general treatment recommendations include immediate induction therapy with 3 or 4 drug regimen with the use of a rifampin sparing agent when possible and consultation with infectious diseases. Then, based on susceptibility data, de-escalation to 2 drug regimen and prolonged treatment for at least 12 months (25).

### Syphilis

Syphilis is caused by the spirochete *Treponema pallidum*. While it is generally sexually transmitted infection, there are other rarer forms of transmission including congenital, *via* blood transfusions, or *via* SOT. As of 2021, the syphilis epidemic continues to be a significant problem and there has been a 68% increase in syphilis cases in the United States since 2017, even in the setting of the global COVID-19 pandemic (39).

Serological testing for syphilis is required prior to transplantation, although in some scenarios, results may not be available until after transplantation. Due to the shortage of donors, a positive test for syphilis is not considered a contraindicated to transplantation, especially given transplant patients can be effectively treated for syphilis with penicillin. If a donor is positive for syphilis, infectious disease specialists should be immediately consulted and appropriate antibiotics should be administered (29).

The preferred treatment for syphilis is long acting benzathine penicillin G administered parenterally. Of particular note, other combinations of penicillin are not considered appropriate treatment and have frequently been inadvertently prescribed (40). Duration of treatment should be discussed with infectious diseases specialists. At times, longer treatment durations are recommended for patients with latent syphilis of unknown duration because the organism may be dividing more slowly (41).

There are no reported cases in the literatures of donor derived syphilis in pediatric kidney transplant patients. There are some published reports of donor derived infection adults, including two adult kidney transplant patients who received kidneys from a common donor whose serologies were found to be positive for syphilis after transplantation. Both were treated for early latent syphilis with the guidance of infectious disease specialists and at 2-year follow-up have excellent kidney function (40). A more recent study found that only 3/25 (12%) syphilis negative recipients who received organs from syphilis positive donors seroconverted at 3 months post-transplant. Of note, those who converted were treponema pallidum particle agglutination negative. None of the kidney recipients seroconverted in this study (42).

### West Nile Virus

West Nile Virus (WNV) is an arborvirus transmitted to humans by way of bites from infected mosquitos, typically of the *Culex* genera, and is the leading cause of mosquito borne disease in the United States (43). In healthy individuals, WNV is most often asymptomatic but the risk for neuroinvasive disease is much higher in the immunocompromised, including DDIs, with high morbidity and mortality (44). The most common presenting symptoms include fever, fatigue, myalgia, and diarrhea (45). Risk of disease correlates with risk of mosquito bites and is highest between May and November (46).

WNV incubation is between 3 and 15 days and methods for diagnosis include serology (IgM and IgG), NAT, or viral culture of serum and/or CSF (24). Infected patients can be viremic for 1–2 weeks. A diagnosis of WNV is made by a positive serum NAT or IgM serology and neuroinvasive disease is confirmed by positive NAT or IgM in the CSF (44). Presence of only IgG in the CSF is likely a false positive and it should also be noted there can be cross reactivity with Japanese Encephalitis, Zika, and Dengue virus. Treatment of WNV is primarily supportive. Current guidelines suggest consideration of intravenous immunoglobulin but note insufficient evidence for use of Interferon *α*-2b or Ribavirin (44).

Donor screening for WNV is not currently required by OPTN policies and practices vary based on center and OPO (47). Current guidelines recommend WNV NAT screening for living donors in endemic areas during months of high WNV. Donor derived WNV can be very difficult to diagnose and require high index of suspicion from the multidisciplinary transplant team for timely management and treatment. Potential living donors who are positive should defer donation for a minimum of 28 days and demonstrate a negative NAT and IgM before proceeding with donation. There are not specific recommendations for screening of deceased donors, although any donor with known WNV infection or positive NAT should be avoided (44). When a donor is infected with WNV, risk of disease transmission to a recipient is high at 87%. Of those who became infected due to donor derived WNC, 30% died or were reported to be in a coma (45).

### Toxoplasmosis

Toxoplasmosis is caused by the parasite *Toxoplasma gondii*, which is considered a neglected parasitic infection by the CDC (48). Worldwide, it is estimated that 30%–50% of the world's population has been exposed to toxoplasmosis, although the infection is frequently asymptomatic, especially in immunocompetent individuals (49). Toxoplasmosis is transmitted through interaction with feces of infected cats, exposure in undercooked meats, and/or on the surface of vegetables grown in contaminated soil. As of 2017, OPTN now requires toxoplasma IgG for all donors (23).

Toxoplasmosis is frequently life threatening in immunocompromised patients. In general, the highest risk for donor derived toxoplasmosis is in heart transplant recipients given the persistence of encysted toxoplasma in the myocardium, but donor derived toxoplasmosis does occur in other SOT. Those who are negative for toxoplasmosis IgG negative prior to transplantation and/or are not on TMP/SMX prophylaxis post-transplant have a higher risk of toxoplasma infection (49).

Usually, donor-derived toxoplasmosis presents in the initial 3 months after transplantation, but has been reported to present as early as day 12 after transplant. The clinical presentation often starts with fever and can progress to multisystem organ dysfunction and disseminated disease, including but not limited to pneumonitis, myocarditis, chorioretinitis, meningitis, brain abscesses (49). There also are multiple reports in the literature of adult kidney transplant recipients who develop hemophagocytic syndrome triggered by toxoplasmosis DDI, which carries a high mortality, up to 60% in some reports (50, 51).

To diagnose active toxoplasmosis, PCR is more sensitive for active infection and samples should be taken of the blood and body fluids. Furthermore, a biopsy of the involved tissue is recommended to identify tachyzoites (this is the rapidly growing life stage of the parasite). General treatment recommendations include induction with pyrimethamine, sulfadiazine, and leucovorin for a minimum of six weeks followed by lifelong suppression in conjunction with infectious disease consultation (49).

### Chagas disease (American trypanosomiasis)

Chagas disease is caused by the protozoan parasite, *Trypanosoma cruzi*. The majority of infections occur in endemic areas of South and Central America. In immunocompetent individuals, most infections are mild with fever or minimal symptoms. Without treatment, the infection is lifelong and can manifest later as severe gastrointestinal and/or cardiac disease in 20%–30% of patients (49). Chagas is transmitted directly by the bite of an infected triatome bug, at childbirth from an infected mother to child, *via* infected food or drink, or blood transfusions, especially in endemic areas (49, 52). OPTN does not require Chagas screening for all donors but does offer geographical guidelines (36).

In non-endemic regions, such as the United States, it is recommended that deceased donors with epidemiological risk factors are screened with serological assay (49). The FDA considers a single positive result with an approved test to be sufficient for the diagnosis of Chagas disease (53). Risk factors include those who were born, received a blood transfusion or lived in an endemic area, whose mother was born in an endemic area, and prolonged stays in rural areas especially with more primitive housing. Of note, sensitivity of serological screening can be reduced due to hemodilution if a donor has received multiple transfusions (53).

The risk of DDI is highest in heart transplant recipients given the parasites affinity for the heart muscle and it is considered a contraindication to heart transplantation. DDI in adult kidney transplants has been reported between 13%–19% (53). The highest risk of DDI is in the first few months post-transplantation. Donor derived Chagas disease will often presents as fever with hepatosplenomegaly and myocarditis. Prospective monitoring should involve both a parasitological method and PCR. If a donor recipient is found to be positive for Chagas disease, treatment should start immediately with antiparasitic therapy, benznidazole or nifurtimox, and infectious disease should be consulted to assist with management (49).

### Strongyloidiasis

Strongyloidiasis is a soil-transmitted helminth typically contracted by contact with free-living larvae in contaminated soil (54). After coming into contact with skin, larvae enter the body and eventually burrow and lay eggs in the small intestine. Mature larvae can re-infect a human host by borrowing into the intestinal wall or penetrate skin around the anus. Less commonly, person-to-person contact can occur, and immunocompromised patients are at particular risk. The helminth is endemic to Southeast Asia, Central and South America, and Africa. It has also been found in the United States (54).

Strongyloidiasis in the SOT patient can occur as a result of *de novo* acquisition in endemic areas, reactivation of latent infection in the setting of immunosuppression, or as a donor-derived infection. SOT patients are at particular risk for hyperinfection syndrome and disseminated disease. Hyperinfection syndrome is characterized by fever, dyspnea, hemoptysis, abdominal pain, nausea, vomiting, diarrhea, intestinal obstruction, or gastrointestinal bleeding. Complications include septic shock and bacteremia due to translocation of gastrointestinal flora. The mortality rate of hyperinfection syndrome and disseminated disease is 50%–70% (55).

Donor-derived strongyloidiasis most commonly involves donors from endemic regions. In a review of 27 adult cases, the median time from transplant to presenting symptoms was 72 days and death occurred in 9 of the cases (56). Bacteremia was a predictor of mortality. In a separate review detailing CDC experience with seven clusters of donor-derived strongyloidiasis from 2009 to 2013, most donors were born in Latin America and did not undergo donor screening (57). Eleven of 20 DDI cases were symptomatic and two died as a result of infection (57). There have been three cases of strongyloidiasis attributed to DDIs in pediatric SOT recipients in the literature with cases reported in the United States and Saudi Arabia (58).

Common symptoms of strongyloidiasis include gastrointestinal concerns, respiratory symptoms, and rash (56). Eosinophilia is often reported in patients with strongyloidiasis but should not be used as a single criterion for diagnosis and can be misleading, particularly as corticosteroid therapy often decreases eosinophil counts. Diagnosis can be made by positive serology or identification of larvae in the stool, either by direct observation or NAT. In disseminated infection, larvae can also be identified in urine, CSF, respiratory secretions, and other body fluids (55). The treatment of choice is a two-day course of oral ivermectin and that is repeated two weeks later. For hyperinfection, treatment should be given daily until 2 weeks after the last positive stool sample. Albendazole is an alternative option, although cure rates are lower when compared to ivermectin (55).

While screening of donors is not required by current OPTN recommendations, AST guidelines strongly recommend screening both living and deceased donors with serology based on epidemiological risk factors (55). Implementation of donor screening protocol for strongyloidiasis has been shown to be effective in multiple cohorts (57, 59). If found to have a positive serology, it is not recommended that a donor be deferred on this criterion alone, as the infection is easily treatable in either a living donor or recipient potential DDI event (55). However, for pediatric recipients, we would advise exercising caution in accepting a deceased donor with risk factors for strongyloidiasis without available testing and await test of cure in potential living donors.

### Candidiasis

*Candida* is classified as a yeast and there are many potentially pathogenic species of *candida* relevant to the SOT population. The most common is *candida albicans*. C*andidia* normally lives on body and can be found in many locations including the skin, oropharynx, gastrointestinal tract, vagina (60). In certain clinical settings such as SOT, candidiasis can cause severe and life threatening infection. DDI can present as candidemia, perirenal hematoma, infected urinoma, abscess, fungal ball, mycotic aneurysm, or anastomotic rupture (17).

Donor derived candida infection is estimated at 1 : 1,000 for kidney transplant recipients. Many of the infections are attributed to contaminated preservation fluid, although the exact risk of developing donor derived candida infection with contaminated preservation fluid is unknown. The contamination is thought to occur prior to or at the time of organ procurement, with the risk increased in the setting of abdominal viscous rupture (17). Regardless of abdominal viscous rupture, it is recommended that routine cultures of the preservation fluid include both bacterial and fungal cultures to improve detection (61). There are also reports of transmission in donors with candidemia. Candiduria is not considered a contraindication to transplantation, although transplant of donors with untreated candidemia is not recommended (17).

Organs in these settings should be used with caution. Early recognition of candida infection is challenging with potentially life-threatening complications. For example, in *candida albicans* arteritis, there is often no preceding clinical symptoms, no evidence of candiduria and no evidence of candidemia prior to presentation until there is severe hemorrhage from the kidney graft anastomosis site (61). While most cases of *candida albicans* arteritis occur within 20 days of transplantation (61) there is also a report of renal mycotic arterial aneurysm up to 47 days post-transplant while on antifungal prophylaxis with caspofungin (62).

In cases of abdominal viscous rupture or candida positive preservation fluid, cultures from the recipient's blood, urine, and any other clinically relevant sites should be obtained (17). In conjunction with infectious disease consultation, empiric antifungal therapy for a minimum of 2 weeks is recommended. Fluconazole is typically the antifungal therapy of choice with close monitoring of calcineurin-inhibitor levels. Kidney ultrasound with doppler should be performed as a baseline and at day 7 post-transplant. If there is strong suspicion for pathology, CT or MR angiography is the imaging of choice. If a recipient has evidence of an aneurysm, there must be an emergent evaluation for nephrectomy. If the recipient has evidence of abscess, drainage should be strongly considered. If a recipient has clinical or microbiological evidence of DDI, antifungal therapy should continue for at least 4–6 weeks. In the setting of vascular involvement, a minimum of 6 weeks is recommended (17).

### Histoplasmosis

*Histoplasmosis capsulatum* is a dimorphic fungus that lives in the environment, especially in soil with large amounts of bat or bird droppings. It is thermally dimorphic which means it exists as mold in the environment and as yeast in an infected host. It is endemic in central and eastern portions of the United States, especially the Ohio and Mississippi River valleys. Other endemic areas around the world include Central and South America, large portions of Australia, Asia, and Africa. Infection most frequently occurs due to inhalation of spores. In immunocompetent hosts, it frequently results in asymptomatic infections although some develop a self-limiting respiratory infection with fever, fatigue, and cough, especially after a high inoculum exposure. In immunocompromised hosts such as SOT, it often presents as severe, disseminated, life threatening infection and almost any site can be involved (63). Symptoms of histoplasmosis in SOT often include fever, fatigue, dry cough, diarrhea, hepatomegaly, splenomegaly, lymphadenopathy, oral ulcers, or sepsis. Central nervous system and skin involvement are relatively uncommon (64).

Currently, pre-transplantation screening of donors is not recommended due to poor positive predictive value of histoplasmosis serologies and low incidence of disease, even in endemic areas. Reactivation is thought to be uncommon, and most cases of post-transplant histoplasmosis are due to *de novo* infection (65). There are a few reported cases of donor derived histoplasmosis in adult and pediatric SOT, although none specifically in pediatric kidney transplant recipients to date (66). It is currently estimated that incidence of donor derived histoplasmosis to be 1 : 10,000 transplant recipients (17, 66). Clinicians needs to maintain a high index of suspicion for disease, especially if donors were from an endemic area. Of note, histoplasmosis can result in false-positive *Aspergillus* galactomannan tests which can result in delay or confusion regarding the underlying diagnosis (66).

Any living donor with active histoplasmosis should be treated with itraconazole for at least 3–6 months prior to organ donation. At time of organ procurement for deceased donors, organs should be evaluated carefully for granulomas. If granulomas are present, histoplasmosis antigen, antibodies, and fungal histopathology and cultures of the appropriate tissues need to be sent. Evidence of granulomas is not an absolute contraindication to pediatric kidney transplantation (17), but extreme caution should be used when accepting an organ for a pediatric transplant recipient given other alternatives such as continuing dialysis.

Treatment is initially determined based on donor findings and should be determined in conjunction with infectious disease consultation. In a deceased donor, if it is found that histoplasmosis was the cause of death, the recipient needs treatment for possible disseminated histoplasmosis for at least one year. In cases where the donor has positive histology but negative culture, oral itraconazole prophylaxis is recommended for at least 3 to 6 months. If a recipient has mild infection, treatment is oral itraconazole. If a recipient develops moderate to severe infection such as a pulmonary infection or disseminated disease, first line treatment is with liposomal amphotericin B for at least 1–2 weeks before transitioning to oral itraconazole (17, 64). In general, serum and urine histoplasmosis antigen testing should occur every 3 months during the duration of prophylaxis or treatment and in cases of treatment, for at least 1 year after therapy is stopped and until the results are negative. If a recipient requires escalated immunosuppression in settings of rejection, continuing itraconazole prophylaxis for longer periods of time should be considered (65).

### Coccidioidomycosis

Coccidioidomycosis is caused by the dimorphic fungi *Coccidiodes immitis* or *Coccidiodes posadasii* (64). It is found predominately in the southwestern United States, although it has also recently been found in the state of Washington. It is also endemic in parts of Mexico, Central America, and South America. Infection usually occurs *via* inhalation of fungal spores and rarely can be transmitted *via* SOT (67). In immunocompetent individuals, 60% have asymptomatic infections, 39.5% develop isolated pulmonary disease or influenza-like symptoms, and <0.5% develop disseminated infection. In SOT, there is a dramatically increased risk of pulmonary and disseminated disease to other organs including CNS, liver, spleen, heat, kidney, skin, and joints (64, 68).

Reactivation or *de novo* infection are more common etiologies of coccidioidomycosis in SOT than infection *via* transplant allograft (68). There are rare reported cases of DDI, including in adult kidney transplants (69). In cases of donor-derived coccidioidomycosis, mortality has been reported to be between 30%–63% (68). Initiation of preventative treatment in cases of potential DDI appear to be very effective, and review of donor derived coccidioidomycoses transmission from 2005 to 2012, there was no deaths in the SOT patients who received prophylaxis (69).

Outside of endemic areas, universal screening is not recommended for coccidioidomycosis. Some centers prescribe prophylaxis in all SOT recipients who live in areas endemic for coccidioidomycoses. Deceased donors should have serological testing if there is evidence of *Coccidioides* on fungal stains of suspicious lung lesions and there should be at least a consideration of serological testing on any deceased donor from an endemic area. If there is evidence or suspicion for coccidioidomycosis in a deceased donor, recipients need to be started on fluconazole or itraconazole prophylaxis and have baseline *Coccidioides* serological testing. Repeat serologies are recommended if the recipient develops suspicious clinical symptoms, even on prophylaxis. Of note, false negative results are possible in SOT due to immunosuppression and cross reaction with *Histoplasmosis* antigen test can occur (64, 68).

In general, fluconazole or itraconazole are the first line treatment for coccidioidomycosis. In cases of rapidly progressive or severe infection, a lipid formula of amphotericin B needs to be initiated. Of note, fluconazole is the preferred treatment for central nervous system infection given amphotericin B has poor penetration of the blood brain barrier (64). After treatment, prolonged fluconazole prophylaxis is recommended. Optimal duration of prophylaxis is unknown and should be determined in conjunction with infectious disease consultation (17). Duration of prophylaxis is further complicated given serologies in SOT can often be falsely negative due to immunosuppression (68).

### Emerging infections

Zoonotic diseases are those transmitted and shared between humans and animals and account for over 60% of all human pathogens per the World Health Organization (70). The WHO also reports that 75% of emerging pathogens over the past decade are due to zoonotic diseases. As our world becomes more connected, the risk of transmission appears to be continually increasing (71). The COVID-19 pandemic demonstrated the dramatic challenges that new infectious disease processes have on our global community, including in solid organ transplantation (14). As discussed earlier in this review, MDROs are another on ongoing challenge and require high levels of vigilance and ongoing antibiotic stewardship.

It is essential that transplant care providers remain vigilant and up to date on emerging infectious diseases and MDRO. It is also vital to have a broad differential diagnosis for DDIs and consider a broad range of potential pathogens. The solid organ transplant community will need to continue careful consideration of both recipient and donors in the setting of emerging infections and/or pandemics. The urgency of the transplantation may also help with the pre-transplant clinical decision-making process and informed consents prior to the acceptance of a potentially high-risk donor in the setting of emerging infections and/or pandemics.

### General treatment issues in DDIs

It is essential to consider drug-drug interactions whenever prescribing a new medication to a SOT recipient. For example, levels of immunosuppressive medication, including but not limited to tacrolimus, sirolimus, and cyclosporine, need to be monitored carefully given drug-drug interactions with azoles (64, 65).

Furthermore, reduction in immunosuppression needs to be considered in setting of a DDI which must be balanced between the risk of rejection and adequate treatment of the infection. This is obviously not without its risks, especially in endemic fungi, where reduction in immunosuppression can also result in immune reconstitution inflammatory syndrome (IRIS) (64). IRIS can result in severe morbidity and mortality and is often mistaken for treatment failure, leading to delay in diagnosis (64). Most cases have been described in adult SOT, although there is one reported case in a 10-year-old pediatric patient with heart transplantation who developed IRIS as a complication of cryptococcal meningitis (72).

Moreover, there is often lack of definitive evidence to determine treatment courses, especially on topics such as secondary prophylaxis in the SOT population (64). The evidence is frequently even more limited in pediatrics. As always in the field of transplantation, a multidisciplinary approach with guidance from consulting teams such as infectious disease and pharmacy can help determine the optimal management.

## Future directions

With the advent of new technologies and diagnostic tools, there is the potential for earlier and more accurate diagnoses of infection to guide decisions about accepting an organ, understand potential risks of transplantation, determine need for post-transplant prophylaxis, and to guide management of DDIs. One example of new technology shaping SOT infectious disease care is next generation sequencing (NGS) of microbial cell-free DNA. There is evidence that NGS of (cf)DNA may result in de-escalation of antimicrobial therapy, shorten duration of therapy, and more rapidly identify microbes than conventional culture-based methods. This also would result in improved antimicrobial stewardship in the SOT population. However, there are still unanswered questions about the challenges of interpretation in immunosuppressed individuals and further investigation is needed (73).

At our center, we experienced the potential benefit of NGS of (cf)DNA in the case of pediatric kidney transplant recipient with unexpected donor derived microsporidiosis (*Encephalitozoon cuniculi*) approximately 12 weeks status post deceased donor kidney transplantation. Our local OPO was alerted that two other patients who had received organs from same donor had posttransplant infections with *Encephalitozoon cuniculi.* Despite an array of tests to assess for *Encephalitozoon cuniculi,* our patient was only found to be positive on microbial cell-free DNA testing. Strong communication with our OPO and transplant teams led to the recognition of *Encephalitozoon cuniculi* in our pediatric patient prior to the development of fulminant disease. To date, our patient has had no evidence of recurrent microspordiosis and microbial cell free DNA has been used to help track the infection and guide management (74). This scenario exemplifies a successful reporting of DDIs per OTPN policy and the use of new technological advances to improve treatment for SOT.

As always, it is essential that the transplant community continues to engage in high quality research and innovation to develop new technologies, directed therapies, and a more personalized approach for our SOT who remain at high risk of DDIs.

## Conclusion

While DDIs overall rare, the consequences can be devastating, especially in the pediatric kidney transplant population. Prevention is key and clinicians must maintain a high index of suspicion for a possible DDIs, especially in any patient who does not follow the expected post-transplant course. As transplant care providers, we must remain vigilant in staying up to date on emerging infections and highly resistant bacteria. Strong communication between OPOs and transplant teams will always be essential to helping recognize, manage, and/or treat DDIs. Furthermore, new innovative ways to rapidly identify potential pathogens prior to transplantation will be essential to help mitigate risk for candidates.
